# Lupus Vasculitis: An Overview

**DOI:** 10.3390/biomedicines9111626

**Published:** 2021-11-05

**Authors:** Patrizia Leone, Marcella Prete, Eleonora Malerba, Antonella Bray, Nicola Susca, Giuseppe Ingravallo, Vito Racanelli

**Affiliations:** 1 Department of Biomedical Sciences and Human Oncology, “Aldo Moro” University of Bari Medical School, 70124 Bari, Italy; patrizia.leone@uniba.it (P.L.); marcella.prete@uniba.it (M.P.); ele94.malerba@gmail.com (E.M.); antonella.bray94@gmail.com (A.B.); susnic@libero.it (N.S.); 2 Section of Pathology, Department of Emergency and Organ Transplantation, “Aldo Moro” University of Bari Medical School, 70124 Bari, Italy; giuseppe.ingravallo@uniba.it

**Keywords:** vasculitis, systemic lupus erythematosus, lupus vasculitis, small vessel vasculitis

## Abstract

Lupus vasculitis (LV) is one of the secondary vasculitides occurring in the setting of systemic lupus erythematosus (SLE) in approximately 50% of patients. It is most commonly associated with small vessels, but medium-sized vessels can also be affected, whereas large vessel involvement is very rare. LV may involve different organ systems and present in a wide variety of clinical manifestations according to the size and site of the vessels involved. LV usually portends a poor prognosis, and a prompt diagnosis is fundamental for a good outcome. The spectrum of involvement ranges from a relatively mild disease affecting small vessels or a single organ to a multiorgan system disease with life-threatening manifestations, such as mesenteric vasculitis, pulmonary hemorrhage, or mononeuritis multiplex. Treatment depends upon the organs involved and the severity of the vasculitis process. In this review, we provide an overview of the different forms of LV, describing their clinical impact and focusing on the available treatment strategies.

## 1. Introduction

Systemic lupus erythematosus (SLE) is a multifactorial systemic autoimmune disease caused by a loss of tolerance to self-antigens, mainly nuclear antigens (DNA, RNA, and nuclear proteins). It is characterized by aberrant T- and B-cell responses, autoantibody production, and immune complex deposition in tissues with complement activation, inflammation, and irreversible organ damage [1]. SLE can affect any organ system, resulting in a wide range of clinical presentations. One of these is vasculitis, which can occur in approximately 50% of SLE patients and principally involve small vessels; medium-sized vessels can also be affected, whereas large vessel involvement is very rare [2,3]. Lupus vasculitis (LV), also known as lupus vasculopathy, may take many clinical forms dependent on the size of the affected vessels and the sites involved, with prognoses that range from mild to life-threatening [3]. Ninety percent of cases affect the skin. The kidneys, gastrointestinal tract, nervous system (central and peripheral), lungs, and heart can be involved with lower frequency [3,4,5]. LV usually appears during an active disease associated with general inflammatory symptoms (fever, fatigue, and weight loss) and laboratory abnormalities (anemia, a high erythrocyte sedimentation rate, and elevated inflammatory markers) [5].

LV can be associated with the antiphospholipid syndrome (APS) characterized by antiphospholipid antibody positivity (lupus anticoagulant, anticardiolipin antibodies, and/or anti-β2-glycoprotein−1 antibodies) [4,6].

In this paper, we provide an overview of the main clinical, diagnostic, and immunological features of LV, with special attention to their impact on SLE severity and outcome. Moreover, we describe the therapeutic strategies commonly used by clinicians, focusing mainly on the available evidence.

## 2. Pathogenesis

The pathogenesis of vasculitis remains poorly understood, but certainly, complex interactions among the vascular endothelium, inflammatory cells, cytokines, and the autoantibodies and immune complexes play crucial roles. LV is a secondary vasculitis that can manifest as an acute or subacute lupus symptom due to an inflammatory process triggered by the deposition of immune complexes in blood vessel walls [7]. Alternatively, it may develop as an associated comorbidity (steroid-related atherosclerosis) or represent the synergistic pathogenetic consequence of enhanced atherosclerosis in a proinflammatory environment [8]. As an autoimmune disease, SLE is characterized by a loss of tolerance to self-antigens, altered T- and B-cell responses, and the production of autoantibodies.

**Figure 1:**
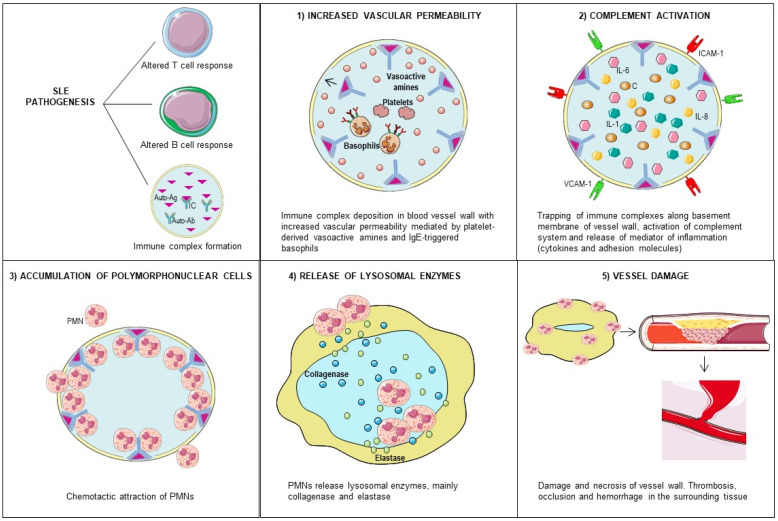
Pathogenesis of lupus vasculitis. Abbreviations: SLE = systemic lupus erythematosus, Ag = antigen, Ab = antibody, IC = immune complex, ICAM-1 = intercellular adhesion molecule 1, VCAM-1 = vascular cell adhesion molecule 1, IL = interleukin, and PMN = polymorphonuclear cell.

The binding of autoantibodies to antigens generates soluble antigen–antibody complexes (immune complexes) that, because of a defective clearance by the reticuloendothelial system and an increase in vascular permeability, are deposited in blood vessel walls. The increased vascular permeability results from the action of platelet-derived vasoactive amines and IgE-mediated reactions [9]. After a complex deposition, the complement system is activated, leading to inflammation and a complement protein consumption [10]. Some complement components act as chemotactic factors for polymorphonuclear leukocytes, which diffusely infiltrate the vessel wall and release their lysosomal enzymes, principally collagenase and elastase, causing damage and necrosis of the vessel wall (Figure 1). This may be associated with thrombosis, occlusion, hemorrhage, and ischemic changes in the surrounding tissue [9]. In addition to the localized increase in vascular permeability, other factors, including the hydrostatic pressure and blood flow turbulence at bifurcations, favor the localization of immune complexes in specific sites of the vascular tree and, thus, the distribution of vascular lesions [11].

Among autoantibodies, anti-endothelial cell antibodies (AECA) are the main cause of endothelial damage. Their binding can induce endothelial cell activation with the upregulation of adhesion molecules (E-selectin, intercellular adhesion molecule 1 (ICAM-1), vascular cell adhesion molecule 1 (VCAM-1)) and the release of cytokines and chemokines, leading to inflammation (interleukin-1 (IL-1), IL-6, and IL-8) [12]; the production of tissue factors favoring coagulation; and the activation of endothelial cell cytotoxicity [13,14]. One study has identified that AECA was positive in more than 80% of the SLE patients [14].

Other types of autoantibodies that may be involved in the pathogenesis of LV are antineutrophil cytoplasmic antibodies (ANCA), anti-phospholipids antibodies (aPL), and anti-double strain DNA (Anti-dsDNA) [15]. ANCA are mainly associated with primary systemic vasculitis, but they also occur in secondary vasculitis linked to systemic connective tissue disorders, including SLE, and may be positive in 15–20% of SLE patients [16,17]. ANCA form immune complexes with proteinase 3 (PR3) or myeloperoxidase (MPO) antigens, leading to neutrophil adhesion to endothelial cells, with a consequent extravascular permeation, vessel damage, and endothelial cell apoptosis [16,17,18]. Focal necrosis of capillaries, venules, and sometimes arterioles occur due to the sequestration of activated neutrophils and monocytes in the microcirculation [18,19]. aPL bind to endothelial cell phospholipids exposed after endothelial damage, causing further endothelial cell damage and activation. Moreover, these antibodies have a role in the complement system activation, resulting in pro-adhesive, proinflammatory, and prothrombotic effects on endothelial cells, and activate endothelial cell thrombin formation by binding to platelet membrane phospholipids [20,21]. Anti-dsDNA induces endothelial cell activation by the stimulation of IL-6 and IL-8 release [22]. They have an anti-endothelial activity, even if a direct cytotoxic effect on endothelial cells has not been established [15].

Changes in cell death pathways, such as apoptosis and the neutrophil-specific kind of death called NETosis, are important contributing factors in the pathogenesis of SLE and LV. An imbalance between the production of apoptotic cells and clearance of apoptotic cells and DNA-containing neutrophil extracellular traps (NETs) can represent a potential source of autoantigens involved in immune complex formation. Immune complexes are cleared by bloodborne macrophages and dendritic cells, resulting in proinflammatory cytokine secretion and the perpetuation of inflammation and tissue damage in SLE patients [23]. NETs are extracellular networks of DNA scaffolds composed of cytosolic and granule proteins that can vary depending on the pathophysiologic context of each disease [24,25]. For instance, the expression of the tissue factor on NETs promotes thromboinflammation in sepsis [26], SLE [27], and vasculitis [28]. In SLE, rheumatoid arthritis and ANCA-associated vasculitis NETs expose immunostimulatory proteins and autoantigens [29].

Some patients with SLE, particularly those with central nervous system involvement, can manifest an inflammatory complement-mediated vascular injury in the absence of immune complex deposition (the Shwartzman phenomenon) [30].

Other forms of vasculitis in SLE patients are drug-induced vasculitis and infection-induced vasculitis [7]. Some drugs, including penicillin, allopurinol, thiazides, pyrazolones, retinoids, cytokines, monoclonal antibodies, chinolons, hydantoin, carbamazepine, and other anticonvulsants, may act as a hapten that, when binding to autoantigens, elicit an immune response, resulting in inflammatory vascular lesions [31,32].

Infection-induced SLE vasculitis may be the result of a direct attack by microbes of the blood vessel wall, followed by an infectious inflammatory process or endothelial cell invasion and activation by certain viruses, e.g., the cytomegalovirus, or the deposition of immune complexes consisting of microbial antigens and their corresponding antibodies [32,33]. In this respect, the association of hepatitis C virus with cryoglobulinemia is remarkable [33,34].

## 3. Cutaneous Vasculitis

**Figure 2:**
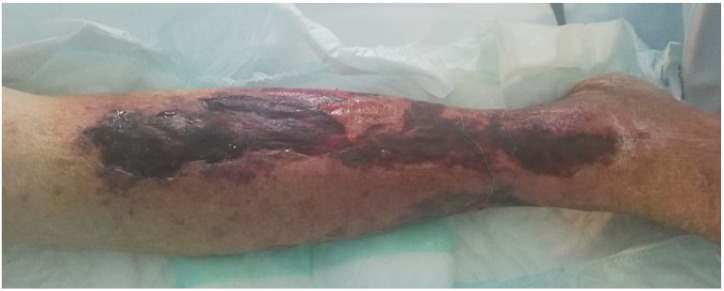
Necrotic purpuric plaques with ulcerations.

**Figure 3:**
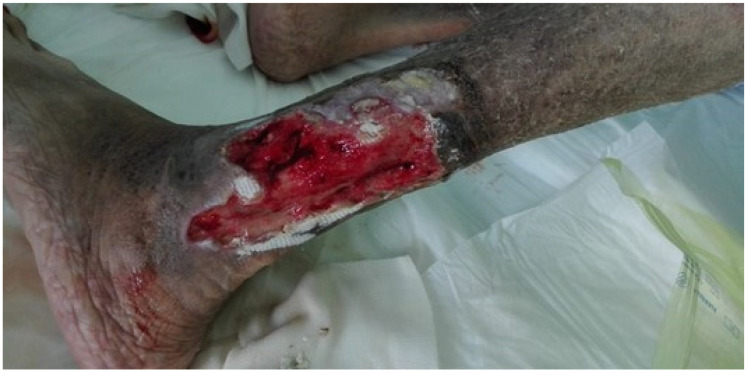
Deep leg ulcer.

Cutaneous vasculitis is the most frequent type of vasculitis among patients with SLE [35]. It is reported in 17–28% of patients with SLE [35,36] and in 89% of the cases of vasculitis in this disease [5]. High levels of anti-Ro and aPL [4,37] and positivity for cryoglobulins [38] are associated with a major risk of developing cutaneous lupus vasculitis. The clinical presentation is heterogeneous and includes palpable purpura, petechiae, papulonodular lesions, urticaria lesions, or bullous lesions of the extremities, livedo reticularis, cutaneous infarction, erythematous plaques or macules, erythema with necrosis, panniculitis, splinter hemorrhages, and superficial ulcerations [4,5] (Figure 2 and Figure 3).

Most skin lesions consist of discoid erythematosus lesions usually located on the fingertips, erythema of the hand dorsum, and nodular lesions [5].

Small vessels, principally post-capillary venules, are involved in most cases. Medium-vessel vasculitis is less frequent and appears as subcutaneous nodules or ischemic ulcers [3]. Myositis and hematological manifestations such as anemia, Coombs’ positivity, leucopenia, anti-Smith, and anti-RNP (ribonucleoprotein) may predict cutaneous vasculitis developments [35].

**Figure 4:**
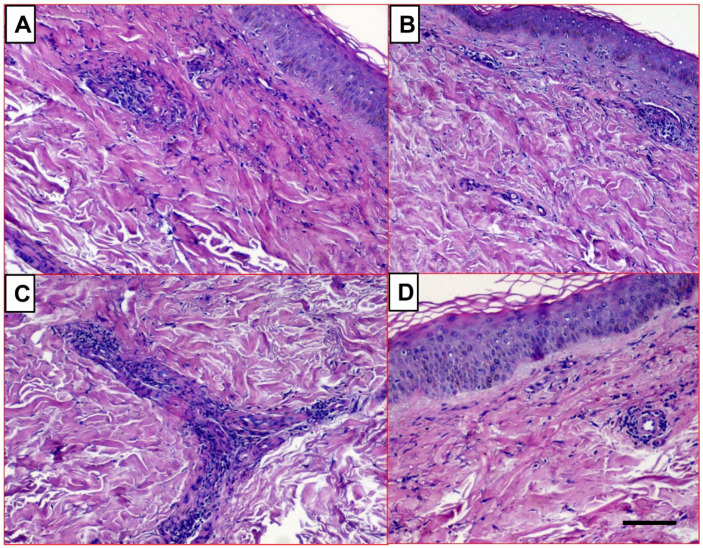
Skin biopsies showing leukocytoclastic vasculitis in SLE patients. The inflammatory lesions are evident in the perivascular derma, associated with nuclear dust and subepithelial myxoid degeneration. (**A**,**B**) original magnification: 40×, scale bar, 50 µm and (**C**,**D**) original magnification: 100×, scale bar, 25 µm.

Skin biopsies from patients with lupus cutaneous vasculitis displayed fragmentated neutrophilic nuclei (a leukocytoclastic variant), dermal chronic inflammation infiltrates, variable fibrinoid necrosis of the vessel walls, and secondary changes in the overlying epidermis and sweat glands (Figure 4).

In two large cohort studies in patients with vasculitis and SLE, the most frequent type of vasculitis was leukocytoclastic vasculitis (60%), followed by cryoglobulinemic vasculitis (25–30%) and urticarial vasculitis (7%) [4,5].

## 4. Nervous System Vasculitis

The nervous system involvement is one of the most complex and heterogeneous features of SLE and occurs more frequently in patients with a high SLE disease activity index. The wide prevalence of neuropsychiatric manifestations ranging between 37% and 95% is due to the difficultly in discerning among the forms due to the disease or to another concomitant process. Noninflammatory microangiopathy in association with brain microinfarctions and thrombosis are common pathogenetic features, whereas inflammatory vasculitis is rare and usually affects the microvasculature [39,40]. Nervous system vasculitis can involve both the peripheral and central nervous systems in SLE patients.

At the peripheral level, the most common clinical manifestation is the mononeuritis multiplex histologically characterized by chronic axonal degeneration, necrotizing, or occlusive vasculitis of the vasa nervorum and demyelination. It affects the individual nerves focally or multifocally rather than many nerves diffusely. The clinical features include pain; weakness; sensory loss; and asymmetrical, progressive, and asynchronous sensory and motor peripheral neuropathy involving at least two separate nerve areas. With disease progression, contiguous nerves become affected, producing a syndrome that mimics a generalized polyneuropathy [41,42]. A mild-to-moderately severe peripheral symmetric sensory polyneuropathy can also develop in SLE patients [43]. Recently, an observational cross-sectional study evaluated the peripheral nerve disease in SLE patients. It found that polyneuropathy was the most frequent manifestation, with an independent statistically significant association with older age at SLE diagnosis and the absence of hematologic involvement as the cumulative SLE manifestation. Three out of nine patients who had undergone a peripheral nerve biopsy showed non-necrotizing small vessel vasculitis [44].

At the central level, cognitive dysfunction, demyelinating syndrome, cerebrovascular disease, and seizure disorders are the most frequently reported clinical features that can appear together or separately in the course of the disease [45]. The diagnosis of central nervous vasculitis is a challenge for clinicians. A brain biopsy is the gold standard for the diagnosis, although it is a highly invasive procedure with a limited sensitivity due to the segmental nature of the vascular lesions. A combination of neuroimaging with clinical features and appropriate diagnostic studies often allows reaching an early diagnosis without a brain biopsy [45]. Actually, the most sensitive noninvasive image study for cerebral SLE-related vasculitis is magnetic resonance imaging (MRI) [45]. However, this technique can reveal wall-thickening and intramural contrast material uptake in vasculitis affecting large brain arteries but does not detect small-vessel involvement [46,47]. Contrast-enhanced MR angiography at 3.0 T and intracranial vessel wall imaging (VWI) modality are also often key supports for a more accurate diagnosis and better differentiation between vasculopathies and intracranial atherosclerotic disease (ICAD). Angiographic imaging can provide information about the vessel lumen and only indirect evidence of vessel wall-thickening. It can show segmental stenosis and dilatation in multiple vascular territories, but these findings are also common in atherosclerotic disease. Moreover, small-vessel disease is beyond angiography resolutions [48]. A cerebrospinal fluid examination does not allow a direct diagnosis of lupus vasculitis but may be the most useful in excluding infections caused by bacterial, viral, and parasitic pathogens [49], as well as autoimmune causes such as multiple sclerosis that can mimic vasculitis [50].

## 5. Gastrointestinal Vasculitis

SLE-related vasculitis of the gastrointestinal tract (also named lupus enteritis) is uncommon; the estimated prevalence varies between 0.2 and 14.2% among all SLE patients [51,52].

A common manifestation of gastrointestinal vasculitis is lupus mesenteric vasculitis (LMV) [53]. It is one of the most devastating complications of SLE, with a mortality rate of 50%, when severe, occlusive damage progresses to bowel ischemia and potential necrosis of the small or large bowel, which may evolve to perforation and hemorrhage [53,54]. In 80–85% of the cases, the superior mesenteric artery is involved, and the structures affected are the ileum and the jejunum; involvement of the large bowel and the rectum is less frequent [55]. LMV often occurs in patients with high disease activity, demonstrated by higher scores on disease activity measurements such as the British Isles Lupus Assessment Group (BILAG) or SLE Disease Activity Index (SLEDAI) [55,56]; altered laboratory data (thrombocytopenia, lymphopenia, hypoalbuminemia, and elevated serum amylase are associated with adverse outcomes); and other coexistent organ involvements, mostly of the skin, kidneys, heart, joints, serositis, lungs, and central nervous system [57]. The main symptoms include acute abdominal pain, nausea, vomiting, diarrhea, melena, hematemesis, and bloating [58]. Urinary symptoms (lupus cystitis and dysuria) can also be associated with LMV in 22.7% of cases [57,59].

The gold standard for diagnosis is computed tomography (CT), which allows visualizing both the bowel wall and the abdominal vasculature. The typical tomographic findings are bowel wall edema, target signs, the dilatation of intestinal segments, prominent mesenteric vessels, increased attenuation of mesenteric fat, and ascites [57,60]. The timely diagnosis of lupus enteritis is crucial to prevent life-threatening complications such as gastrointestinal perforation, hemorrhage, and sepsis [61]. Relapses can occur in 31.7% of cases [62].

Within the abdominal cavity, vasculitis may also affect the liver and pancreas. Necrotizing arteritis of the liver has been reported in 18–20% of SLE autopsy cases [63,64]. Alazani et al. described a hepatic vasculitis mimicking multiple liver abscesses in a patient with SLE, which showed clinical improvement after steroid therapy [65]. Spontaneous hepatic rupture due to small- and medium-sized vessel vasculitis is an unusual complication [66,67]. Although SLE-related acute pancreatitis is uncommon, it is more severe and frequently fatal and should be suspected in SLE patients with abdominal pain [68]. Vasculitis has been related to pancreatitis in a subset of SLE patients [69].

## 6. Renal Vasculitis

Five pathological types of renal microvascular lesions have been described in patients with lupus nephritis (Table 1) [70]. So far, the attention has been mainly focused on glomerular pathology, and renal vascular lesions have been overlooked.

A Chinese study analyzed 341 patients with lupus nephritis and found 279 (81.8%) patients with renal vascular lesions, including 74.2% with immune complex deposition, 24.0% with nonspecific arteriosclerosis, 17.6% with thrombotic microangiopathy, 3.8% with noninflammatory necrotizing vasculopathy, and 0.6% with true renal vasculitis. Approximately 40% of the cases presented with more than two types of vascular lesions [71].

True renal vasculitis is the least frequent renal vascular injury found in lupus nephritis and has been infrequently reported in the literature. It was found retrospectively in 2.8% [72], 2.4% [73], and 0.6% of renal biopsies [71]. True renal vasculitis can be morphologically differentiated from the other, more frequent forms of vascular renal lesions in SLE given that it is the only form yet described in which there is true inflammatory infiltration of the intima and media [71].

More attention should be dedicated to the patterns of renal microvascular lesions. The presence of vascular in a on lupus nephritis biopsy is associated with a worse prognosis and the risk of end-stage renal disease (ESRD) but not independent of the serum creatinine and nephritis class [71,74,75,76]. Histological lesions can present as glomerular, tubulointerstitial, and microvascular lesions and can affect small- and medium-sized arteries, most commonly intralobular arteries [69]. Mural inflammation with prominent inflammatory cell infiltrates and fibrinoid necrosis may be found [77]. Although clinical presentations vary with different types of vascular lesions, in general, SLE patients with renal vasculitis manifest with glomerular lesions, severe hypertension that likely worsens the vascular changes, anemia, hematuria, severe renal insufficiently with rapid progression to renal failure, and high SLEDAI scores [70].

A diffuse proliferative glomerulonephritis is considered by some authors as the most frequent form of renal vasculitis involving glomerular capillaries. However, the general agreement is that these lesions should be classified as proliferative lupus glomerulonephritis [78].

## 7. Retinal Vasculitis

Retinal vasculitis is a very uncommon complication documented in few case reports. Although the exact pathogenesis is unclear, it is thought that immune complex deposition, complement activation and aPL play a role [79]. Typically, the precapillary superficial arterial vasculature is involved [80]. Retinal vasculitis can present as asymptomatic or with painless blurred vision, decreased vision, or even permanent visual loss. A funduscopic examination reveals retinal vessel sheathing, cotton wool spots, retinal hemorrhage, and vascular occlusion [81]. Retinal imaging, including fluorescein angiography and optical coherence tomography, can be helpful in the identification and characterization of retinal vasculitis [81,82]. Using multimodal imaging techniques and electrophysiology, Chin et al. recently described a rare case of severe bilateral lupus retinal vasculitis associated with paracentral acute middle maculopathy. Both the superficial and deep retinal capillary vasculature was involved, resulting in marked generalized retinal dysfunction. The combination of immunomodulatory therapy with localized pan-retinal laser photocoagulation has led to an improvement in vision, the prevention of neovascularization, and remission of SLE [80].

## 8. Coronary Vasculitis

Coronary vasculitis is a rare condition with few case reports published in the literature. There is no strong association between SLE clinical activity and coronary arteritis. It often manifests in the absence of clinical SLE flare and laboratory evidence of active SLE [39]. The diagnosis is usually made by serial coronary angiographic studies that disclose arterial aneurysms, tapered stenoses, and/or rapidly developing arterial occlusions [69]. Histopathologically, coronary artery thrombosis or immune complex deposits, with an infiltration of lymphocytes and neutrophils, and fibrinoid necrosis can be detected [83,84]. Rare examples of cardiac valve dysfunction and myocardial dysfunction due to small vessel vasculitis have also been reported [69].

## 9. Pulmonary Vasculitis

**Figure 5:**
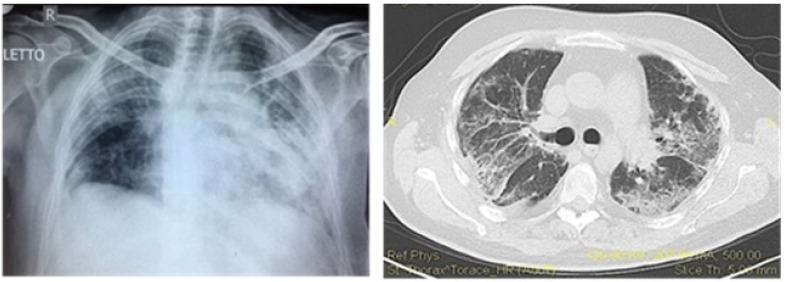
Diffuse alveolar hemorrhage. A chest radiography shows bilateral widespread infiltrates in both medium lower fields. A chest computed tomography shows left pulmonary embolism and massive diffuse infiltration of both lung fields. R means right.

The most common clinical manifestation of lupus pulmonary vasculitis is diffuse alveolar hemorrhage (DAH) due to the access of red blood cells within the alveolar spaces as a result of the widespread damage of the pulmonary vessels with disruption of the alveolar-capillary basement membrane [85]. The imaging studies often describe classical bilateral alveolar interstitial infiltrates (Figure 5).

It usually occurs in the context of high disease activity and could be a severe complication of SLE with a mortality rate of 35.3% [86]. Symptoms may include cough, progressive and severe dyspnea, fever, chest pain, and hemoptysis in over 60% of cases. Some patients may be asymptomatic [87]. The diagnosis is based on CT, a chest radiography, bronchoscopy, and bronchial lavage. Histopathologically, capillaritis and mononuclear infiltrates, alveolar necrosis, and immune complex deposits of IgG and C3 can be found [39].

## 10. Association between Lupus Vasculitis and Antiphospholipid Syndrome

SLE is the most common disease with which APS occurs [20], and within SLE patients, there is an association between LV and APS. Vascular injury and APS are often present simultaneously in SLE patients [88,89,90] and are closely connected with each other given that aPL can contribute to the damage of vascular endothelium during the vasculitic process [20,21].

Specifically, aPL plays a pathogenetic role in some forms of LV, including retinal vasculitis [79], DAH [91,92,93], and renal vasculitis [70]. The concomitant presence of vasculitis and APS is associated with a poor outcome. In particular, a comparison of the renal disease severity and outcome in patients with primary APS, APS secondary to SLE, and SLE alone revealed that APS worsens the prognosis of lupus nephritis [94].

## 11. Treatment

The treatment of LV is extremely varied and requires a timely diagnostic framework to limit the potentially severe consequences and life-threatening manifestations. The wide spectrum of clinical manifestations as a result of the inflammatory involvement of different-sized vessels and different organs is the main limiting factor in the management of these patients. Mesenteric vasculitis with bowel ischemia [52]; nervous systemic vasculitis—specifically, multiple mononeuropathy, seizures, and transverse myelitis [95]—and pulmonary vasculitis as diffuse alveolar hemorrhage [96] are the main severe complications in SLE that need timely aggressive therapy.

Unfortunately, no robust body of literature is available to guide their management, and therapeutic recommendations are commonly based upon other autoimmune conditions or case reports, case series, and expert opinions [97] (Table 2).

In this context, the updated EULAR (European League Against Rheumatism) recommendations for SLE are a clinical reference point, although LV treatment should be tailored to the severity of the disease and its associated symptoms [127]. Mild-to-moderate manifestations are usually handled with oral corticosteroids and immunosuppressants such as methotrexate, azathioprine, and mycophenolate mofetil. A more aggressive therapy with intravenous high-dose corticosteroids, cyclophosphamide, rituximab, intravenous immunoglobulin, and/or plasmapheresis is considered for the severe and life-threatening forms [69].

Cutaneous vasculitis often requires anti-malarias; hydroxychloroquine (200–400 mg/day) has been considered in some patients with success, primarily those with hypocomplementemia urticarial vasculitis [98,128]. It is generally well-tolerated, though its potential ophthalmological toxicity is well-known and needs regular monitoring. In skin-limited vasculitis, the responses to colchicine (0.6 mg twice daily) have been described in several open-label case series [99,100,101], although relapses have been reported after colchicine therapy interruption. In the case of poor efficacy or contraindications for the drug, thalidomide and dapsone (50–200 mg/day) can be used, with good results [97,102,128]. In a multicenter Chinese study, 69 patients with cutaneous lesions of SLE were treated for 8 weeks with a starting dose of thalidomide at 25 mg daily and gradually increased. The maximum ratio of an effective and maintenance dose of thalidomide were both at 50 mg daily, and the rate of total remission rose to 100% at the eighth week [102]. Oral glucocorticoids (methylprednisolone > 15 mg/day) may be required for a short period of time for painful, ulcerative, or otherwise severe diseases in order to speed up the resolution [1,97,129]. Among Conventional Disease-Modifying Antirheumatic Drugs (cDMARDs), azathioprine (2 mg/kg/day) has been successfully used in the treatment of various types of systemic vasculitis, including severe lupus cutaneous vasculitis resistant to conventional therapy, with some adverse events such as leukopenia, hepatic injury, hypersensitivity reaction, and infections [97,100,103]. For resistant cases of lupus cutaneous vasculitis, immunoglobulin could be an off-label option. The usual starting dose is 1 g/kg for 2 consecutive days, followed by 400 mg/kg monthly until disease resolution or for 6 months [104,105]. The anti-CD20 antibody rituximab is a safe, effective treatment for refractory chronic cutaneous small vessel vasculitis that is nonresponsive to traditional therapies [106]. Although there are no data about the effectiveness of JAK inhibitors in LV, the high efficacy of these drugs in many skin manifestations, including atopic dermatitis [130], psoriasis [131], and graft-versus-host disease [132], suggests that they may represent a new effective weapon for treating LV.

The current treatment for gastrointestinal LV includes a high dose of corticosteroids, intravenous infusions of methylprednisolone with subsequent tapering, and for patients with recurrent disease or that do not respond to intravenous prednisolone alone, intravenous cyclophosphamide should be considered [57,58,107,108,109,133]. The successful use of rituximab has also been reported in case series [107]. When a rapid response is not achieved, surgical options should be considered.

In a controlled long-term clinical trial, multiplex mononeuropathy, seizures, and transverse myelitis were successfully treated with a frontline therapy based on methylprednisolone 3 g daily for 3 days as the induction treatment, followed by cyclophosphamide for 2 years at 0.75 g/m^2^ of the body surface, monthly for 1 year, and then every 3 months for another year [95]. Several studies have supported the off-label use of rituximab in cases of severe refractory neuropsychiatric SLE, but a relapse after rituximab treatment was observed in 45% of the cases [110].

The treatment of renal microvascular lesions in lupus nephritis remains undefined, and the current therapeutic strategies are based on glomerular pathology. The gold standard for inducing remission in systemic necrotizing vasculitis and severe lupus nephritis is the combination of high-dose steroids with cyclophosphamide or mycophenolate mofetil as the induction therapy and low-dose steroids combined with varying regimens of azathioprine or mycophenolate mofetil for the maintenance phase [111,112,134]. An update of a Cochrane review first published in 2004 showed that a mycophenolate mofetil treatment can result in increased complete disease remission compared with cyclophosphamide, with acceptable toxicity, although with low certainty evidence. Calcineurin inhibitors, alone and in combination with mycophenolate mofetil, may have comparable or improved rates of disease remission compared with cyclophosphamide and a lower toxicity but uncertain effects. Maintenance therapy based on azathioprine may increase the disease relapse compared with mycophenolate mofetil [112]. A retrospective study assessed the efficacy of plasmapheresis in patients with lupus nephritis combined with thrombotic microangiopathy, highlighting the improvements in recovery and renal outcome in patients who received plasmapheresis associated with corticosteroid and immunosuppressive drugs compared with those treated with corticosteroid and immunosuppressive drugs alone [113]. The role of rituximab in the induction therapy has not been clearly established for lupus nephritis. In a recent systematic review, an analysis of 31 studies of rituximab for class I-VI lupus nephritis revealed the heterogeneous efficacy of rituximab alone or in combination with cyclophosphamide or mycophenolate mofetil among patients of different ethnic and racial backgrounds, lupus nephritis classes, time courses of the disease, ages, and prior immunosuppressive uses [114]. For cases resistant to conventional therapy, alternative strategies may be used. Eculizumab, a fully humanized monoclonal antibody that inhibits the human C5 complement component, might be an alternative treatment of severe refractory lupus renal vasculitis [115,135]. Recently, new drugs, including Obinutuzumab (anti-CD20 monoclonal antibody) for B-cell depletion or belimumab (anti-”B-cell activating factor” monoclonal antibody neutralization) for B-cell neutralization and Voclosporin (a calcineurin inhibitor with a low profile of renal and systemic toxicity) have shown promising results regarding an improvement in the renal response in addition to the standard therapy in patients with lupus nephritis and might represent new potential therapeutic strategies for lupus renal vasculitis [136,137,138].

In pulmonary vasculitis with DAH, a high dose of methylprednisolone (4–8 g, above the conventional dosage of 3 g) [96] and cyclophosphamide remain the most commonly used therapies [139,140]. Plasmapheresis [116] and rituximab [86] are the other beneficial treatment options in refractory cases [92]. Experimental strategies such as Intrapulmonary Factor VII therapy [117], extracorporeal membrane oxygenation [118], and umbilical cord-derived mesenchymal stem cell transplantation [119] are limited to selective severe cases.

The mainstay of the treatment of retinal vasculitis in lupus is systemic immunosuppression with high-dose oral corticosteroids. Once disease control is reached, low-dose systemic glucocorticoids and hydroxychloroquine are often used long term to prevent disease flares. Cases of severe retinal vasculitis that are refractory to steroid therapy are treated with plasmapheresis; rituximab; or a combination of plasmapheresis, rituximab, and intravitreal ranibizumab (a monoclonal antibody against vascular endothelial growth factor A) [120,121,122,123]. In pediatric patients with SLE and occlusive retinopathy, an early intervention with a combination of B-cell depletion therapy and a traditional cytotoxic agent such as cyclophosphamide should be considered [124]. Pan-retinal photocoagulation and intravitreal anti-vascular endothelial growth factor injections can induce the regression of macular edema and retinal neovascularization [80,124].

There is no established therapy for SLE coronary vasculitis, although few case reports have reported clinical benefits with intravenous pulse dose methylprednisolone associated with intravenous cyclophosphamide in active SLE patients [83,125,141]. When SLE coronary vasculitis is refractory to immunosuppressant therapy, an orthotropic heart transplant should be performed [126].

## 12. Conclusions

Vasculitis occurs frequently in SLE patients with an active disease and poor prognosis. Lupus vasculitis is characterized by varying manifestations, given that it can affect any organ system. A prompt diagnosis and adequate treatment are essential. The modalities for treatment are tailored according to the presentation and severity of the disease. Due to a lack of randomized controlled trials specific for LV, therapeutic decisions are based on experiences treating other autoimmune conditions, other forms of vasculitis syndromes, or case reports.

## Figures and Tables

**Table 1:** Pathological types of renal microvascular lesions.

Uncomplicated vascular immune deposits	Immune deposits in the wall of small renal arteries without inflammation, necrosis, or thrombosis are more commonly associated with active glomerular proliferative forms of lupus nephritis. By the light microscopy examination of renal biopsy specimens, the normal histology is assessed. By immunofluorescence microscopy, staining for IgG, IgA, IgM, and various complement components (often C1q or C3) can be observed in the vessel wall. By electron microscopy, the deposits are electron dense, with a granular texture, and are most commonly observed below an intact vascular endothelium or within the basement membranes.
Arteriosclerosis	It is characterized by an increased arterial wall thickness and reduction of the vascular lumen due to fibrotic intimal thickening and replication of the internal elastic lamina.
Noninflammatory necrotizing vasculopathy	It may be considered a complication of more severe forms of immune complex deposition. The immune complex deposits can cause luminal narrowing or occlusion and are accompanied by necrotizing damage, frequently found in preglomerular arterioles and less in interlobular arteries. Abundant glassy eosinophilic materials may occupy the lumen and intima and, sometimes, may extend into the media. The endothelium is usually swollen or denuded, and the elastic membrane is often disrupted. The inflammatory infiltrate is rare. IgG, IgM, and IgA positivity can be detected by immunofluorescence microscopy in the vessel wall and in the lumen, as well as complement components and fibrin-related antigens. By electron microscopy, swelling or loss of the endothelium can be seen along with abundant intraluminal and mural deposits of granular electron-dense materials.
Thrombotic microangiopathy	It is most frequent in SLE patients with thrombotic thrombocytopenic purpura or anticardiolipin syndrome. In the early phase, there is swelling of the endothelial cells and subendothelial space. During the acute phase, a severe narrowing or total occlusion of the arteriolar lumen may be found. Fibrinoid necrosis may also be detect. The chronic phase presents swelling of the intima of the interlobular arteries associated with mucoid intimal edema and/or “onion skin” pattern lesions as result of the cellular intimal proliferation. By immunofluorescence microscopy, fibrinogen or fibrin in the walls of arterioles and small arteries can be observed, as well as IgM, IgG, IgA, C3, and C1q positivity. Electron microscopy may highlight the swelling and detachment of the endothelium from the underlying structures and an expanded intima.
True renal vasculitis	It is the least common renal lupus vascular lesion that usually involves small arteries, most commonly intralobular arteries. Histologically, it is indistinguishable from the polyarteritis nodosa. Morphologically, these lesions are characterized by neutrophils and mononuclear leukocytes that eccentrically or circumferentially infiltrate the intima and media. In the acute phase, this infiltration is often associated with fibrinoid necrosis and rupture of the elastic lamellae. Immunofluorescence reveals strong staining for fibrin-related antigens, with weak and variable staining for immunoglobulin and the complement.

**Table 2:** The treatments of different vascular manifestations in SLE.

Vascular Manifestation	Type of Study	No. of Patients	Treatment	% Response (or Remission)	Adverse Events	Ref.
Cutaneous	Case report	1	Hydroxychloroquine (200–400 mg/day)	100	N.R.	[98]
	Case series	13	Colchicine (0.5–0.6 mg twice daily)	69	Mild (adominal cramping and diarrhea)	[99]
	Clinical experience	10		70	Mild	[100]
	Prospective randomized controlled trial	41		29	Mild	[101]
	Multicenter study	69	Thalidomide (50 mg/day)	100	Mild (drowsiness and constipation)	[102]
	Case series	6	Azathioprine (2 mg/kg/day)	33	Leukopenia, hepatic injury, hypersensitivity reaction, and infections	[103]
	Clinical trial	12	Immunoglobulin (1 g/kg for 2 consecutive days followed by 400 mg/kg monthly)	>75	N.R	[104]
	Case report	1			Patient died from septic shock	[105]
	Case series	2	Rituximab	100	N.R.	[106]
Gastrointestinal	Retrospective cohort study	97	Cyclophosphamide (500–1000 mg/m^2^) and prednisone (>30 mg) Mycophenolate mofetil (2 g/day) Corticosteroid only	84.5	Severe adverse events occurred in 15 patients	[57]
	Retrospective study	38	Methylprednisolone (1 mg/kg/day)	100	N.R.	[58]
	Case series	5	Cyclophosphamide and corticosteroids	80	N.R.	[107]
			Rituximab	20		
	Case series	3	Methylprednisolone (20 mg/kg/day for 5 days) and cyclophosphamide (1 g/m^2^)	100	N.R.	[108]
	Case series	19	Methylprednisolone pulse therapy (1 g/day for 3 days), followed by cyclophosphamide (1 g/m^2^ intravenously) in 4 cases.	90	N.R.	[109]
Nervous system	Controlled clinical trial	32	Cyclophosphamide (0.75 g/m^2^ monthly for 1 year and then every 3 months for another year). Methylprednisolone (1 g daily for 3 days, monthly for 4 months, then bimonthly for 6 months and subsequently every 3 months for 1 year) Oral prednisone on the fourth day of treatment (1 mg/kg/day)	75	Infections of the gastrointestinal, urinary and upper respiratory tract. Herpes zoster	[95]
	Case report and review of 34 cases	35	Rituximab and concomitant treatment with corticosteroids, methylprednisolone, cyclophosphamide or azathioprine	50	Herpes zoster Infections	[110]
Renal	Meta-analysis (18 studies)	1989	Mycophenolate mofetil and cyclophosphamide	N.R.	Infections	[111]
	Meta-analysis (74 studies)	5175	High-dose steroids with cyclophosphamide or mycophenolate mofetil as induction therapy, and low-dose steroids combined with varying regimens of azathioprine or mycophenolate mofetil for the maintenance phase	N.R.	Diarrhea	[112]
	Retrospective study	61	Cyclophosphamide or mycophenolate mofetil in combination with glucocorticoids for the induction phase. Mycophenolate mofetil or azathioprine combined with low-dose glucocorticoid regimens for the maintence phase.	N.R.	No severe adverse events	[113]
		9	Plasmapheresis and baseline immunosuppressive therapy	33	None	
	Systematic review (31 studies)	1259	Rituximab alone or in combination with cyclophosphamide or mycophenolate mofetil	77 Caucasian 38 East-Asian 28 Hispanic	N.R.	[114]
	Systematic review (15 studies) and case report	20	Eculizumab	85	N.R.	[115]
Pulmonary	Case series	16	High-dose corticosteroid, followed by pulse methylprednisolone, plasmapheresis, pulse cyclophosphamide, and rituximab	N.R.	Infections	[86]
	Case series	34	High dose of methylprednisolone (>3 g) and cyclophosphamide	N.R.	N.R.	[96]
	Retrospective clinical trials	40 SLE (11 DHA)	Therapeutic plasma exchange	N.R.	Mild (bleeding)	[116]
	Case control study	22	Various combinations of corticosteroids, plasmapheresis, cyclophosphamide, rituximab, and mycophenolate mofetil.	N.R.	N.R.	[92]
	Case report	1	rFVIIa 75 μg/kg	N.R.	None	[117]
	Case report	1	Extracorporeal Membrane Oxygenation	N.R.	None	[118]
	Retrospective study	4	Umbilical cord-derived mesenchymal stem cell transplantation	N.R.	None	[119]
Retinal	Case report	1	Plasmapheresis and the bilateral administration of intravitreal ranibizumab (0.5 mg) and rituximab	N.R.	None	[120]
	Case report	1	Plasmapheresis, followed by rituximab and mycophenolate mofetil	100	None	[121]
	Case report		Rituximab (1 g) and cyclophosphamide (10 mg/kg)	100	None	[122]
	Case series	2	Plasmapheresis, followed by a single intravenous infusion of cyclophosphamide (750 mg) Plasmapheresis and methotrexate (15 mg weekly)	N.R.	None	[123]
	Case series	2	Panretinal photocoagulation, rituximab (750 mg/m^2^ × 2 weeks), and cyclophosphamide (750 mg/m2 per dose) with a concurrent pulse of methylprednisolone (1000 mg) Methylprednisolone (1000 mg) plus rituximab (750 mg/m^2^ × 2 weeks) and monthly cyclophosphamide (750 mg/m^2^ × 7 months).	N.R.	N.R.	[124]
Coronary	Case report	1	Prednisone (60 mg once a day) and cyclophosphamide (1300 mg monthly)	100	N.R.	[83]
	Case report	1	Methylprednisolone (1 g) and cyclophosphamide (1 g)	100	N.R.	[125]
	Case report	1	Methylprednisolone 1000 mg daily for 3 days, followed by 1-mg/kg/day prednisone in addition to a single dose of intravenous cyclophosphamide 860 mg and oral hydroxychloroquine 400 mg daily. Orthotopic heart transplant	0	N.R.	[126]

Abbreviations: N.R. = not reported; DHA = diffuse alveolar hemorrhage; rFVIIa = recombinant activated coagulation factor VII.
